# A Psychometric Evaluation of the Body Image Questionnaire Child and Adolescent Version

**DOI:** 10.1007/s10578-024-01768-1

**Published:** 2024-10-15

**Authors:** L. Blacker, M. Gupta, R. Quinn, B. Monzani, A. Jassi, D. Veale, D. Mataix-Cols, G. Krebs

**Affiliations:** 1 Research Department of Clinical, Educational & Health Psychology, University College London, 1–19 Torrington Place, WC1E 7HB London, UK; 2 Department of Psychology, Institute of Psychiatry, Psychology & Neuroscience, King’s College London, London, UK; 3 National and Specialist OCD, BDD and Related Disorders Clinic for Young People, South London and Maudsley NHS Trust, London, UK; 4 Centre for Anxiety Disorders and Trauma, South London and Maudsley NHS Foundation Trust, London, UK; 5 Department of Clinical Neuroscience, Centre for Psychiatry Research, Karolinska Institutet & Stockholm Health Care Services, Region Stockholm, Stockholm, Sweden; 6 Department of Clinical Sciences, Lund University, Lund, Sweden

**Keywords:** Body dysmorphic disorder, Adolescents, Assessment, Psychometric, Factor analysis

## Abstract

Body dysmorphic disorder (BDD) typically develops during adolescence, but there has been little research evaluating assessment tools for BDD in youth. This study sought to provide a comprehensive psychometric evaluation of a brief self-report questionnaire of BDD symptoms, the Body Image Questionnaire Child and Adolescent version (BIQ-C), in both clinical and non-clinical adolescent samples. Properties of the BIQ-C were examined in 479 adolescents recruited through schools and 118 young people with BDD attending a specialist clinic. Sensitivity to change was additionally examined in a subgroup of the clinical sample who received treatment (*n* = 35). Exploratory factor analysis indicated that a two-factor structure provided the best fit for the data in the non-clinical sample. The two-factor solution was corroborated through confirmatory factor analysis as the best solution in the clinical sample, although it did not fulfil predefined fit thresholds The first factor encompassed preoccupation and repetitive behaviours, while the second included items assessing functional impairment. The BIQ-C showed good internal consistency across both samples, and convergent validity with other measures of BDD. Among those in the clinical sample who received treatment, BIQ-C scores decreased significantly, and BIQ-C change scores were highly correlated with change scores on the gold-standard clinician-rated measure of BDD symptom severity. These findings indicate that the BIQ-C is a suitable tool for assessing BDD symptoms in young people and measuring change during treatment.

**Supplementary Information:**

The online version contains supplementary material available at 10.1007/s10578-024-01768-1.

## Introduction

Body dysmorphic disorder (BDD) is a complex and potentially debilitating condition, affecting approximately 2% of the population [17, 40]. The disorder typically emerges during adolescence [3], although often goes undetected and undiagnosed for many years. Underdiagnosis of BDD may partly result from sufferers often avoiding mental health services due high levels of shame and a preference for cosmetic treatments. Even among those who do attend mental health services, BDD may be overlooked due to diagnostic overshadowing [33] and a lack of awareness of BDD among clinicians [37]. Consequently, ensuring the timely identification and assessment of BDD in clinical settings is an important clinical priority [13].

The most widely accepted tool for assessing BDD symptoms in youth is the adolescent version of the Yale-Brown Obsessive–Compulsive Scale Modified for Body Dysmorphic Disorder (BDD-YBOCS-A). Previous research has highlighted its strong psychometric properties including, a two-factor structure accounting for 56% variance, strong internal consistency (Cronbach’s alpha = 0.87), and adequate convergent and divergent validity [22]. However, the BDD-YBOCS-A is a clinician-administered measure, requiring both significant time commitment and specialist training. Self-report measures present a more cost-effective and efficient way to identify those with BDD symptomology, thereby informing clinical assessment [38]. Similarly, self-report measures are an efficient way of measuring symptom change during a course of treatment. This is crucial at an individual level, to inform ongoing care planning, but also enables evaluation of treatment efficacy and effectiveness in research and service contexts, respectively. Currently, self-report measures of BDD symptoms in youth are scarce and are either lengthy and only validated in non-clinical samples [31] or focus specifically on cognitive and behavioural process relevant to therapy and do not capture distress and impairment [14].

The Body Image Questionnaire (BIQ) (also called the Cosmetic Procedures Screening, or COPS, when used in cosmetic settings) is a self-report measure to assess BDD symptoms in adults [38]. The BIQ is widely used in research and clinical settings. For example, it is the BDD symptom measure that is used in NHS Talking Therapies for Anxiety and Depression (formally known as Increasing Access to Psychological Therapies, or IAPT), the largest provider of psychological therapy in the United Kingdom [35]. The BIQ was originally developed as a 12-item scale but subsequently shortened to a 9-item version The 9-item version is the most widely utilised in research and clinical settings as it has been found to have high sensitivity and superior psychometric properties [32, 38]. The psychometric properties of the BIQ (9-item) have been evaluated in adult samples, demonstrating strong internal consistency and convergent validity, and a single-factor structure [38]. The BIQ has also been adapted for use in children and adolescents but there has been limited research into the properties of the youth version of the BIQ (referred to as the Body Image Questionnaire – Child and Adolescent,BIQ-C. Establishing reliable and valid measures of BDD symptoms in youth is crucial, since BDD typically develops during adolescence and may be associated with more severe and negative outcomes compared to later onset BDD [3, 23].

To our knowledge, there has only been one previous psychometric evaluation of the BIQ-C, and this focused on establishing the factor structure and assessing measurement invariance across sex [32]. A two-factor structure was established and partial scalar invariances across sexes, suggesting that the BIQ-C scores can be compared between boys and girls [32]. The first factor included items relating to interference and avoidance, and the second factor comprised ‘other BDD symptoms’ which included items assessing preoccupation, appearance-related compulsions and distress. However, it should be noted that these findings are derived from a non-clinical adolescent sample recruited through schools, not young people with BDD. In line with methodological best practice [4, 21, 26]. There is a need to evaluate the BIQ-C in young people with BDD, as this represents the population for which the BIQ-C was originally developed. [4, 21, 26]. Furthermore, there is a need to establish the broader range of psychometric properties of the BIQ-C, such as convergent and divergent validity and sensitivity to change.

The current study sought to fill the gaps stated above, through a comprehensive evaluation of the psychometric properties of BIQ-C in a non-clinical sample of adolescents recruited through schools and a clinical sample of adolescents with a confirmed diagnosis of BDD attending a specialist clinic. In line with evidence from previous psychometric studies, we chose to focus on evaluation of the 9-item version of BIQ-C [32, 38]. More specifically, we aimed to determine the factor structure of the BIQ-C, internal consistency, convergent and divergent validity, and treatment sensitivity. We did not have an a priori hypothesis regarding the factor structure of the BIQ-C. However, we did anticipate that the BIQ-C would have high internal consistency, good convergent and divergent validity (as indicated by a higher correlation with other measures of BDD symptoms than with measure of other aspects of psychopathology) and would be sensitive to change over treatment.

## Methods

### Participants

The non-clinical sample comprised of 479 young people aged between 14 and 18 years, recruited through secondary schools in London to take part in survey-based studies [15, 18]. Young people completed a battery of questionnaires, including the BIQ-C. The clinical sample was made up of 118 young people with a confirmed primary diagnosis of DSM-5 BDD (American Psychiatric Association, 2022), attending the Maudsley specialist OCD, BDD and Related Disorders Clinic [22]. The majority attended the service as part of routine clinical care (*n* = 91, 77.1%), and the remainder participated in a randomised controlled trial (RCT) of cognitive behaviour therapy (CBT) for BDD (*n* = 27, 22.9%) [20]. Within the overall clinical sample, 35 patients (29.7%) had post-treatment BIQ-C data available for analysis, having received a full course of CBT for BDD according to a validated protocol and/or pharmacotherapy [20, 28]. The remaining patients (*n* = 87, 73.7%) were either referred elsewhere for treatment, did not complete treatment or did not have post-treatment data available (e.g., currently in treatment or did not complete post-treatment questionnaire).

### Measures

#### The Body Image Questionnaire – Child and adolescent version (BIQ-C)

The BIQ-C is a self-report measure of severity of BDD symptoms [38]. The first question, not included in the final BIQ-C score, asks about the area(s) of appearance concern. This is followed by 9- items, which contribute to a final score and cover core symptoms of BDD including preoccupation, repetitive behaviours, distress, and interference. Items are rated on a 0 to 8 Likert scale, with reverse scoring of items 2, 3 and 5, yielding a total score ranging from 0 to 72. A cut-off score of 40 is applied to indicate clinically significant symptoms [38]. The BIQ-C is adapted from the adult BIQ. Adaptations comprised changing the wording of some items to increase relevance for young people (e.g., referencing interference with school rather than interference with work). In adult community and BDD samples, the BIQ demonstrated strong internal consistency (α = 0.91) and convergent validity with measures related to depression (*r* = 0.70), anxiety (*r* = 0.66) and body image related quality of life (*r* =—0.68) [38]. In a non-clinical adolescent sample, the BIQ-C also illustrated strong internal consistency across genders (female α = 0.89, male α = 0.84) [32]. The BIQ-C was completed by both non-clinical and clinical samples.

#### The Yale-Brown Obsessive–Compulsive Scale modified for Body Dysmorphic Disorder Adolescent version (BDD-YBOCS-A)

The BDD-YBOCS-A is a 12-item clinician-administered measure of BDD symptom severity [25]. It asks questions related to preoccupation, compulsive behaviours, insight and avoidance behaviours. Each item is rated on a scale from 0 to 4, generating a total out of 48, with higher scores indicating greater symptom severity. Scores of 24 or more, indicate clinical case-ness [39]. In adolescent samples the measure demonstrated good internal consistency (α = 0.87), reasonable convergent validity with self-report BDD measures (the Appearance Anxiety Inventory (*r* = 0.37, p < 0.01)), and significant negative correlation with the Children’s Global Assessment Scale (*r* = −0.57, p < 0.01) reflecting the negative impact BDD symptoms can have on functioning [22]. Similar psychometric properties are recorded in adult clinical samples [24]. The BDD-YBOCS-A was completed by the clinical sample.

#### The Appearance Anxiety Inventory (AAI)

The AAI is a 10-item self-administered measure, which measures BDD-related cognitions and behaviours [39]. Each item is rated on a scale from 0 to 4, with a higher score indicating greater severity or impairment, generating a maximum total score of 40. The AAI has demonstrated good internal consistency in clinical (α = 0.86) and non-clinical (α = 0.91–0.97) samples and adequate sensitivity to treatment [14, 30, 39]. The measure also shows significant convergent validity and a positive correlation with clinician-administered measures of BDD symptoms (*r* = 0.42, *p* < 0.001) [14, 39], measures of appearance related sensitivity (Body Dysmorphic Concerns Questionnaire *r* = 0.74, *p* < 0.001) and measures of social anxiety (*r* = 0.53, *p* < 0.001) [30, 39]. The AAI also correlates negatively with measures of quality of life (r =−0.54, *p* < 0.001) [39]. The AAI was completed by the non-clinical and clinical samples.

#### The Children’s Global Assessment Scale (CGAS)

The CGAS is a single-item, clinician administered, measure assessing global functional impairment resulting from psychopathology [34]. It is rated between 0 and 100, with higher scorings indicating higher levels of functioning. The measure shows good psychometric properties with high interrater reliability between administering specialists and across time. In addition, it illustrates strong discriminant validity and significant convergent validity [9, 34]. The CGAS was completed by the clinical sample.

#### The Mood and Feeling Questionnaire Child version (MFQ-C)

The MFQ-C is a 33-item, self-report, measure, which assesses levels of depressive symptoms in young people aged between 6 and 19 years old [2]. The measure illustrated strong internal consistency in clinical adolescent samples (α = 0.94) and high criterion validity [6, 41]. The MFQ was completed by the clinical sample.

#### The Strengths and Difficulties Questionnaire (SDQ)

The SDQ is a 25-item, self-report, or informant-report measure. Items are spread across five scales assessing behaviours relating to conduct problems, hyperactivity, emotional symptoms, peer problems and pro-social behaviour. Respondents review statements and indicate if they are “Not True”, “Somewhat True” or “Certainly True”. Responses are scored from 0 to 2, with several items reversed scored. Higher scores indicate a higher level of negative behaviours. The self-report sub-scales illustrated good psychometric properties but could not be used as a diagnosis tool [12]. The SDQ was completed by the clinical sample.

#### The Revised Children’s Anxiety and Depression Scale (RCADS)

The RCADS is a self-report measure, assessing anxiety and depression-related symptoms in young people. The baseline version contains 47-items across six sub-scales: separation anxiety disorder, social phobia, generalised anxiety disorder, panic disorder, obsessive compulsive disorder, and low mood (major depressive disorder) [7]. Shorter versions have been developed, including the 11-item and 25-item versions validated in non-clinical samples (*n*(11-item) = 177, *n*(25-item) = 302) [10, 27], which collapse items into two core-subscales: anxiety and low mood. Items are scores on a 4-point Likert scale: 0—“Never”, 1—“Sometimes”, 2—“Often” or 3 – “Always”. Higher scores indicate more severe anxiety or depressive symptoms. All three versions of the scale, illustrate acceptable to good psychometric properties across community and clinical samples [7, 10, 27]. The RCADs was completed by the non-clinical sample.

#### The Child-Adolescent Perfectionism Scale (CAPS)

The CAPS is a 22-item, self-report measure assessing perfectionism [11]. The measure is split into two sub-scales: socially prescribed perfectionism, and self-oriented perfectionism. Items are scored on a 5-point Likert scale, with 0 indicating a statement is “False” and 5 indicating the statement is “True”. The measure has been found to have good psychometric properties across heterogenous community samples [11]. The CAPS was completed by the non-clinical sample.

### Procedure

Participants for the non-clinical sample were recruited for two survey-based studies from government-funded schools in South London, UK, and aged from 14 to 18 years. Participants completed the measures digitally (full details of the procedure have been previously described [15, 18]). Ethical approval was provided in each case from the Psychiatry, Nursing and Midwifery Research Ethics Subcommittee of King’s College London (HR-16/17–3877 and HR/DP-20/21–2139) [15, 18].

Participants in the clinical sample were assessed by a specialist multidisciplinary team, which included administration of the BDD-YBOCS-A. Self-reported measures were collected prior to the initial assessment and at post-treatment (see [28]for further details). Diagnoses of BDD were arrived at by a multidisciplinary team, according to DSM-5 criteria (American Psychiatric Association, 2022), leveraging information collected via the BDD-YBOCS-A interview, a separate parent interview, and the developmental history. A total of 35 patients (29.7%) completed a course of treatment for BDD, either as part of routine clinical care or as participants in an RCT of CBT for BDD and completed the BIQ-C post-treatment. Treatment comprised CBT with or without concomitant medication. CBT consisted of weekly sessions incorporating psychoeducation, exposure and response prevention, and relapse prevention, as previously described [20, 28]. Psychometric evaluation of the BIQ-C in the clinical sample was approved by the South London and Maudsley Child and Adolescent Mental Health Service Audit Committee as described by Monzani et al. [22]. Ethical approval for the RCT was granted by the National Research Ethics Service Committee South East Coast – Kent (REC reference 11/LO/1605) [20].

### Statistical analyses

The analysis protocol was pre-registered on the Open Science Framework (OSF) (https://osf.io/kmd34). Effort was made prior to analysis, to determine if the available samples were of sufficient size to enable a threshold power of 0.8, as detailed in the (see Table S1 of supplement). Our psychometric evaluation was composed of four elements: factor analysis; internal consistency calculation; assessment of convergent and divergent validity; and assessment of treatment sensitivity.

The factor analyses were composed of an exploratory factor analysis (EFA) for structure definition, followed by validation via confirmatory factor analysis. The EFA was conducted in the non-clinical sample and utilised R package psych [29]. EFA was conducted in the first instance as prior research into the factor structure of the BIQ-C and COPS had produced varied results, and there was limited theoretical basis from which to pre-specify a factor structure. All 479 subjects from the non-clinical sample were included in the EFA analysis. The sample’s suitability for factor analysis was assessed prior to analysis, first score distributions were inspected for non-linearity, followed by testing for multicollinearity between items. Item correlations of *r* > 0.90 were utilised as a threshold for item exclusion. A Kasier-Meyer-Olkin test was then performed, followed by Bartlett’s test for sphericity. The number of factors to extract was determined through inspection of the scree plot, combined with results of a parallel analysis. Factor extraction was then completed, using a maximum likelihood extraction method and a Promax rotation. Resulting factor loadings were inspected to assess factor model fit.

Following completion of the EFA in the non-clinical sample a clearer view of factor structure was produced, which mirrored existing literature. Consequently, in line with best practice [21, 26], a confirmatory factor analysis (CFA) was undertaken in the clinical dataset. As before, suitability of the sample for factor analysis was assessed prior. The analysis sought to test the fit of the two-factor model generated in the EFA and compare it with a one-factor model. Model fit for both models was evaluated utilising Bayesian Information Criterion (BIC), Comparative Fit Index (CFI) and Root Mean Squared Error of Approximation (RMSEA) metrics. Model fit was deemed superior where |ΔBIC|> 2 when compared with the alternate model, CFI >  = 0.95 indicated a good fit, and RMSEA < 0.05, or 0.05 < RMSEA < 0.08, indicative of close and reasonable fit respectively.

Across both samples, internal consistency (IC) was evaluated using Cronbach’s alpha (with α = 0.70 acceptability threshold applied). IC was calculated at three levels: the overall measure, within-factor, and within-factor if item excluded. Item-total correlations (ITC) were calculated within factor, to evaluate the influence of specific item scores on the patterns in total scores.

Convergent/ divergent validity was assessed through examination of Pearson’s correlation coefficients comparing total BIQ-C score to total scores on comparison measures. Statistical comparison of BDD-related measures was completed using Fischer’s r to z transformation. Within the non-clinical sample, the BIQ-C total score was compared with total AAI, CAPS and RCADS scores. In the clinical sample, the BIQ-C total score was compared with total scores on the AAI, BDD-YBOCS-A, MFQ, SDQ and CGAS.

Finally, treatment sensitivity was evaluated in the clinical sample via paired t-test and calculation of within-group effect size (Cohen’s *d*) comparing BIQ-C scores pre- and post-treatment. These were compared to the treatment sensitivity the BDD-YBOCS-A.

For all measures other than the BIQ-C, if participants had completed less than 20% of items, the missing items were imputed utilising mean substitution. In circumstances where missing data exceeded 20% per measure, participants were excluded from relevant analyses. In the non-clinical sample, two versions of the RCADS were used (i.e. an 11-item and a 25-item version), and so z-transformations were applied to total RCADS scores to enable harmonisation of the datasets.

## Results

### Participant characteristics

Characteristics of the non-clinical and clinical samples are shown in Table 1. Across both samples, but particularly the clinical sample, a majority of participants were female. Ages ranged from 14 to 18 and 11 to 18 years old across the non-clinical and clinical samples respectively, with the non-clinical sample having a greater mean age. White British ethnicity was also dominant across both samples, accounting for 19.2% and 48.3% of the non-clinical and clinical samples respectively.

**Table 1:** Study sample characteristics

		Non-clinical sample M(SD) or n(%)	Clinical sample M (SD) or n(%)	Group comparison t-test (t) or chi-squared (χ)
n		479	118	–
Age	Age	16.4 (0.6)	15.9 (1.4)	*t*(123.55) = 3.53, *p* < .001
Gender	Female	265 (55.3%)	80 (67.8%)	χ^2^ (3) = 50.95, *p* = < .001
	Male	203 (42.4%)	24 (20.3%)	
	Other	6 (1.3%)	0 (0.0%)	
Ethnicity	White British	92 (19.2%)	57 (48.3%)	χ^2^ (6) = 52.66, *p* = < .001
	White Other	26 (5.4%)	4 (3.4%)	
	Asian or Asian British	12 (2.5%)	1 (0.8%)	
	Black or Black British	22 (4.6%)	2 (1.7%)	
	Mixed Background	17 (3.5%)	7 (5.9%)	
	Other Ethnic Groups	8 (1.7%)	5 (4.2%)	
	Not specified	302 (63.0%)	42 (35.6%)	
Measure scores	BIQ – C (9-item)	21.8 (14.2)	54.0 (12.2)	*t*(202.78) = −24.81, *p* < .001
	AAI	10.9 (9.0)	28.8 (7.4)	*t*(213.37) = −22.59, *p* < .001
	CAPS	26.5 (7.7)	–	–
	RCADS-25 RCADS-11	16.6 (11.6) 14.5 (8.3)	–	–
	BDD-YBOCS-A	–	32.7 (6.1)	–
	CGAS	–	42.4 (8.9)	–
	MFQ	–	40.4 (14.7)	–
	SDQ	–	26.2 (5.4)	–

Two different versions of the RCADS were used in non-clinical participants, and therefore two means (and standard deviations) are presented. For subsequent analyses, RCADS scores were z-transformed to enable harmonisation of the data

As expected, the mean total BIQ-C score was significantly lower in the non-clinical sample than the clinical sample. The prevalence of clinically significant BDD symptoms (BIQ-C score ≥ 40) was 13.4% in the non-clinical sample. The mean AAI score was also significantly lower in the non-clinical sample relative to the clinical sample. In the clinical sample, the mean BDD-YBOCS-A score was 32.7 (*SD* = 6.1), indicating moderate to severe symptoms. Mean scores on the CGAS indicated a moderate degree of interference in functioning in most social areas or severe impairment in functioning in one social area.

### Factor structure

#### Exploratory factor analysis

Prior to factor analysis, item-level correlations were inspected, which demonstrated sufficient levels of correlation between items in the BIQ-C. This was further supported by the KMO, which indicated adequate sampling (0.90) and a significant result from Bartlett’s Test for Sphericity (χ^2^ (36) = 1972.23, *p* < 0.001). The number of factors to extract was determined to be between one and three factors (see Figure S1 of supplement). Based on Kaiser’s rule (eigenvalue > 1) a one factor structure was identified, however this method of factor identification is not considered robust, it was therefore used in conjunction with inspection of the scree plot and parallel analysis using principal axis factoring, which indicated a two factor and three factor structure respectively.

Each permutation of factor number was fit to the data, using a maximum likelihood estimation procedure and Promax rotation. Comparison of factor structures indicated that the two-factor structure was the most parsimonious factor structure (Table 2), which mirrored the two-factor structure defined by Scheider et al. (2018). The three-factor structure was discounted, as one of the three factors only had a single item loaded onto it. The two-factor structure for the BIQ-C accounted for 53% of the variance (Factor 1—30%, Factor 2—23%). Factor loadings are detailed in Table 3.

**Table 2:** BIQ-C item factor loadings for non-clinical sample, and item-total correlations and Cronbach’s alpha scores for non-clinical (*n* = 479) and clinical (*n* = 118) samples

	Factor loadings (non-clinical sample)		Item-total correlation (ITC)^a^		Cronbach’s alpha^b^	
Item	Factor 1 (Preoccupation)	Factor 2 (Impairment)	Non-clinical sample [95% CI]	Clinical sample [95% CI]	Non-clinical sample [95% CI]	Clinical sample [95% CI]
How often do you check your feature(s)?	0.79	−0.20	0.58 [0.52, 0.64]	0.43 [0.27, 0.57]	0.83 [0.80, 0.85]	0.79 [0.72, 0.85]
How much do you feel your feature(s) is ugly, unattractive or ‘not right’?	0.66	0.01	0.60 [0.54, 0.65]	0.50 [0.35, 0.62]	0.82 [0.79, 0.84]	0.74 [0.66, 0.81]
How much does your feature(s) cause you a lot of distress?	0.64	0.18	0.69 [0.64, 0.73]	0.63 [0.50, 0.73]	0.80 [0.76, 0.82]	0.71 [0.61, 0.78]
How often does your feature(s) lead you to avoid places or activities?	0.18	0.43	0.51 [0.44, 0.57]	0.65 [0.53, 0.74]	0.77 [0.73, 0.80]	0.73 [0.63, 0.80]
How much is your feature(s) on your mind? That is, you think about it a lot and it is hard to stop thinking about it?	0.72	0.14	0.74 [0.70, 0.78]	0.74 [0.64, 0.81]	0.78 [0.75, 0.81]	0.68 [0.57, 0.76]
If you have a girlfriend or boyfriend, how much does your feature(s) have an effect on your relationship with him or her? OR If you do not have a girlfriend or boyfriend but would like one, how much does it have an effect on you getting one?	0.24	0.41	0.52 [0.45, 0.58]	0.43 [0.27, 0.57]	0.76 [0.73, 0.80]	0.82 [0.76, 0.87]
How much does your feature(s) get in the way with your school or college work?	0.05	0.73	0.66 [0.60, 0.71]	0.59 [0.46, 0.70]	0.71 [0.66, 0.75]	0.75 [0.66, 0.82]
How much does your feature(s) get in the way with your social life (i.e. spending time with friends, going to parties)?	-0.02	0.88	0.70 [0.65, 0.74]	0.77 [0.69, 0.84]	0.66 [0.61, 0.71]	0.65 [0.53, 0.75]
How much do you feel your appearance is the most important thing about you?	0.55	0.16	0.62 [0.56, 0.67]	0.54 [0.40, 0.67]	0.82 [0.79, 0.84]	0.73 [0.64, 0.80]

^*a*^*ITC* = *Correlation coefficient for correlation between total score minus item score and item score (per factor)*

^*b*^*Cronbach’s alpha* = *internal consistency if item removed (per factor)*

**Table 3:** Internal consistency overall and per factor across clinical and non-clinical samples

	Non-clinical	Clinical
Total measure	0.88 (95% CI [0.87, 0.90])	0.83 (95% CI [0.78, 0.87])
Factor 1	0.84 (95% CI [0.82, 0.86])	0.77 (95% CI [0.70, 0.83])
Factor 2	0.78 (95% CI [0.74, 0.81])	0.80 (95% CI [0.73, 0.85])

#### Confirmatory factor analysis

The two-factor solution was then tested via CFA in the clinical sample. This was compared with a one-factor solution. The fit indices indicated the two-factor solution (BIC = 4195.16, CFI = 0.85, RMSEA = 0.15 (90% CI [0.11, 0.18])) was comparatively superior to that of the one-factor (BIC = 4254.28, CFI = 0.71, RMSEA = 0.20 (90% CI [0.17, 0.23]), although test statistics for both one- (χ^2^ (27) = 154.52, *p* < 0.001) and two- (χ^2^ (26) = 90.63, *p* < 0.001) factor models, indicated neither model achieved fit threshold.

### Internal consistency

Cronbach’s alpha for the pre-treatment BIQ-C measure was 0.88 and 0.83 for the non- clinical and clinical samples respectively, both indicating good internal consistency. Cronbach’s alpha for the post-treatment BIQ-C in the clinical sample was 0.89. Internal consistency of the two factors was good across both samples (Table 3). Item-total correlations exceeded 0.30 and ranged between 0.40 and 0.77 (Table 2) across both samples, indicating that each item contributed sufficiently to the corresponding factor.

### Convergent and divergent validity

The BIQ-9 showed significant convergent validity with the AAI across both non-clinical and clinical samples, as indicated by correlation coefficients ranging from 0.76 – 0.86 in both samples (see Table 4). In the non-clinical sample, the BIQ-C had a larger correlation with the AAI than the RCADS (anxiety and depressive symptoms) or CAPS (perfectionism), indicating relative divergent validity with these measures. Similarly, in the clinical sample, the BIQ-C had a larger correlation with the AAI than any other measure, again demonstrating divergent validity with these measures. Of note, the BIQ-C showed a moderate correlation with the clinician-rated BDD-YBOCS-A (Table 4).

**Table 4:** Convergent and divergent validity evaluated by Pearson correlation coefficients across both clinical and non-clinical samples

	Non-clinical	Clinical
AAI	0.86 [0.83, 0.88]***	0.76 [0.67, 0.82]***
RCADS	0.65 [0.59, 0.70]***	–
CAPS	0.33 [0.25, 0.41]***	–
BDD-YBOCS-A	–	0.59 [0.45, 0.70]***
MFQ	–	0.63 [0.48, 0.74]***
CGAS	–	−0.57 [-0.68, -0.42]***
SDQ	–	0.42 [0.24, 0.56]***

95% confidence intervals in parentheses

***p < .001

### Sensitivity to change

Thirty-five patients in the clinical sample completed the BIQ-C both at pre-treatment and post-treatment. There was a significant reduction in BIQ-C score between these two time points, with mean scores of 54.0 (SD = 11.6) and 35.9 (18.6) at pre-treatment and post-treatment respectively (*t*(34) = 5.58, p < 0.001). Within group effect size (Cohen’s *d*) for the BIQ-C from pre-treatment to post-treatment was large (*d* = 1.17, 95% CI [0.66, 1.69]). The change in BIQ-C scores were also highly correlated with BDD-YBOCS-A change scores (*r* = 0.69, [95% CI 0.45, 0.84], p < 0.001).

## Discussion

This study represents the first comprehensive psychometric evaluation of the BIQ-C in a community sample of adolescents and a clinical sample of young people with BDD. A two-factor structure for the BIQ was identified in the non-clinical sample and confirmed in the clinical sample. The separation of BIQ items onto the two factors aligned exactly with the findings of Schneider et al. in a community sample of adolescents [32]. One of the factors involved items relating to preoccupation and repetitive behaviours, for example the frequency of feature checking and amount of time dedicated to thinking about the feature. The other factor was comprised of items assessing functional impairment resulting from BDD symptoms, for example avoidance of activities or people, and impact on schooling and friendships. These groupings correspond with the core features of BDD, as set out in the DSM-5 [1]. In addition, the results of the CFA indicated levels of covariance between items relating to peer relationships, school, and social activities, which may support suggestions that concerns relating to peer-perception of physical appearance and social functioning are particularly salient to adolescents [19].

Of note, the current findings did not align with the one-factor structure found by Veale et al. in an adult sample [38]. The difference may be explained by the differences in the scope and statistical approaches across the two studies. For example, Veale et al. focussed on adult populations sourced from a cosmetic procedures clinic whereas this study focussed on adolescents across community and clinical psychiatric services. Veale et al. [38] also utilised a principal component analysis (PCA) method, while this study adopted a factor analysis (FA) approach. FA was selected over PCA in the current study in keeping with the COnsensus-based Standards for the selection of health Measurement INstruments (COSMIN) guidelines, and because it seeks to explain covariances between items, rather than the cumulative variance, and therefore provides an estimation of the unmeasurable latent factors upon which the measure is based [16]. It is important to note that in the current study neither the one- or two-factor structures met strict thresholds for ‘good fit’ according to fit indices, although the two-factor structure was superior.

In the current study, the BIQ-C demonstrated good internal consistency in both non-clinical and clinical samples, when considered as a whole measure, and when considered across the two factors. This indicates that items within the test are homogenous, delivering a cohesive scale that measures the same underlying construct. When considering the impact of discrete items on internal consistency, items assessing social impairment with peers had greatest positive influence, suggesting that social functioning is particularly salient feature of BDD in adolescence. This aligns with conventional wisdom that adolescents place greater weight on the opinions of their peers [5], therefore, individuals with BDD may be more likely to avoid peer interactions, for fear of judgement of the perceived flaw [36].

We found the BIQ-C to have good convergent validity, as demonstrated by large correlations with the AAI, another self-report measure assessing behavioural and cognitive processes associated with BDD. In the clinical sample, the BIQ-C was also highly correlated with the BDD-YBOCS-A. Of note, the correlation of the BIQ-C with the BDD-YBOCS-A was smaller than that with the AAI, which is likely to due to the fact the BIQ-C and AAI are self-report whereas the BDD-YBOCS-A is clinician-rated. As hypothesised, we found evidence of divergent validity, demonstrated by lower correlations of the BIQ-C with non-BDD measures. Nevertheless, the correlations of the BIQ-C with self-reported measure of depressive and/or anxiety symptoms were substantial across both samples, which may be due to overlap in phenomenology as well as genuine comorbidity. In the clinical sample, the BIQ-C also demonstrated a large, negative correlation with the CGAS, consistent with previous research showing the profound functional impairment caused by BDD in youth [28].

The BIQ-C was also found to be appropriate for measuring changes in BDD symptom severity over the course of treatment. Scores decreased significantly from baseline to post-treatment, evidencing sensitivity to change. This was further supported by highly correlated change scores between the BIQ-C and the BDD-YBOCS-A (*r* = 0.69). The results compare favourably to the sensitivity to change of the AAI, the only other BDD self-report measure that has been evaluated in young people receiving treatment for BDD. Previous research has found changes in AAI scores over treatment to be moderately correlated (*r* = 0.55) with changes in BDD symptom severity measured by the BDD-YBOCS-A [14] The larger correlation observed for the BIQ-C may reflect the fact that this measure captures distress and impairment, as does the BDD-YBOCS-A, whereas the AAI focuses on cognitive and behavioural processes associated with BDD. When considered together, the findings illustrate the suitability of the BIQ-C as a measure to assess treatment outcomes in adolescents with BDD in clinical practice and in research contexts.

To expand upon our findings, future studies should seek to establish clinical cut-offs for detecting the presence of clinically significant BDD symptoms. It is notable that in the current study, 13% of the community sample scored above the adult-derived BIQ cut-off score for clinically significant symptoms. This is much higher than the prevalence of BDD among adolescents in the general population, which has been estimated at 1.9% [17]. The elevated prevalence in our community sample may in part reflect selection bias (i.e. those who self-identified as having appearance anxiety were more likely to take part in the study). However, it is also possible that the adult-derived cut-off for clinically significant symptoms is not applicable in adolescent samples, and a higher threshold is required to differentiate clinically significant BDD symptoms from normative adolescent appearance concerns. It is also possible that the BIQ-C captures broader body image issues beyond BDD, such as eating disorder psychopathology and concerns relating to real visible differences in appearance (e.g. acne). Further research is needed to determine the extent to which the BIQ-C can differentiate BDD symptoms from other forms of body image problems, which will be crucial in determining its utility as a BDD screener. Additionally, future research should seek to determine severity cut-offs on the BIQ-C for differentiating mild versus moderate versus severe symptoms, which could assist in clinical decision-making.

### Strengths and limitations

This pre-registered study represents the first comprehensive psychometric evaluation of the BIQ-C in non-clinical and clinical adolescent samples. A best practice approach for factor analysis applied, namely EFA followed by validation via CFA [4]. Nevertheless, there were limitations to the current study. The number of factors to extract within the EFA, varied depending on extraction technique, from one to three factors. This was mirrored in the CFA where neither the one or two factor structures met relevant fit thresholds. This may have been due to insufficient power in the sample or may illustrate some instability in the BIQ-C measure, which may require further analysis.

Another limitation is that different measures were given to the clinical and non-clinical sample, except for the AAI which was administered in both samples. The limited overlap in measures precludes the possibility of comparing associated symptoms (e.g. of depression and anxiety) across the two groups There was also a significant amount of data missing in the clinical post-treatment dataset, for both BIQ-C and BDD-YBOCS-A, which may have introduced bias into the treatment sensitivity analyses. Additionally, the clinical sample cannot be considered fully representative of the whole BDD population that meet clinical threshold. The sample was taken from a specialist service, where there are high levels of comorbidity and treatment-resistance, thereby impacting the generalisability of results produced. Lastly, we were not sufficiently powered to test measurement invariance across demographic groups (e.g. gender, ethnicity, age), and future studies should seek to address this question.

### Summary

This study represents the first comprehensive psychometric analysis of the BIQ-C in clinical and non-clinical adolescent samples. We found evidence supporting a two-factor structure, good internal consistency, good convergent validity with BDD-related measures, and robust sensitivity to change. Our findings therefore support its use in clinical settings and research settings.

## Supplementary Information

Below is the link to the electronic supplementary material.

Supplementary file1 (DOCX 87 KB)

## Data Availability

Data may be made available upon request, and subject to appropriate data sharing agreements.
