# Supplementary material for: A Psychometric Evaluation of the Body Image Questionnaire Child and Adolescent Version
Source: Child Psychiatry Hum Dev. 2024 Oct 15;57(4):1037–47. doi: 10.1007/s10578-024-01768-1 (PMC13437764; doi:10.1007/s10578-024-01768-1)
Supplement: Supplementary file 1 [file 10578_2024_1768_MOESM1_ESM.docx]

**A psychometric evaluation of the Body Image Questionnaire Child and Adolescent version**

**Supplementary Information**

# **Table S1: Output of Power Analysis calculations**

| **Statistical Analysis** | **Power Analysis Results** |
| --- | --- |
| Factor Analysis | There is no agreed methodology for power calculations for factor analysis (MacCallum et al., 1999), although the proposition of a minimum ratio of n (sample size) f (number of factors) of approximately 10 : 1 is commonly cited (Everitt, 1975). Work by Veale et al. (2012) indicated a one-factor structure of the BIQ in a sample of n = 108 adults (Veale et al., 2012) and work by Schneider et al. (2018) indicate a two-factor structure in a sample of n = 1,512 young people (Schneider et al., 2018). Based on the rule above these factor structures require a minimum of 10 and 20 participants respectively, both our clinical and non-clinical samples contain >100 participants, indicating we should have sufficient power for a factor analysis. |
| Internal Consistency (Cronbach’s alpha) | Utilising approaches defined in previous literature (Bonett & Wright, 2015; Bujang et al., 2018) we estimated a minimum sample size of 14 (where power = 0.8, significance level = 0.05, threshold Cronbach’s Alpha = 0.7 and Cronbach’s alpha for null hypothesis = 0). Our clinical and non-clinical samples are therefore of sufficient size to determine a reliable estimate of Cronbach’s alpha. |
| Convergent/ divergent validity | Utilising the R package pwr (Champely et al., 2017),, a minimum sample size of 85 was calculated (where power = 0.8, significance level = 0.05 and Pearson’s correlation coefficient = 0.3), meaning all our samples are of sufficient size to estimate this validity measure. |
| Sensitivity to change | Utilising the R package pwr (Champely et al., 2017),, a minimum sample size of 34 was determined to enable detection of a medium effect size of 0.5 (where power = 0.8, significance level = 0.05 and type = “paired”). As above, this means both our clinical and non-clinical samples of sufficient size to estimate this sensitivity measure. A medium effect size is deemed suitable as previous work into treatment efficacy indicates a within-subject effect size of well over 1.0 (Krebs et al., 2017; Mataix-Cols et al., 2015; Rautio et al., 2022) |

# **Figure S1: Scree Plot and Parallel Analysis output BIQ-C (9-item)**

# **References**

Bonett, D. G., & Wright, T. A. (2015). Cronbach’s alpha reliability: Interval estimation, hypothesis testing, and sample size planning. *Journal of Organizational Behavior*, *36*(1), 3–15. https://doi.org/10.1002/job.1960

Bujang, M. A., Omar, E. D., & Baharum, N. A. (2018). A Review on Sample Size Determination for Cronbach’s Alpha Test: A Simple Guide for Researchers. *The Malaysian Journal of Medical Sciences : MJMS*, *25*(6), 85–99. https://doi.org/10.21315/mjms2018.25.6.9

Champely, S., Ekstrom, C., Dalgaard, P., Gill, J., Weibelzahl, S., Anandkumar, A., Ford, C., Volcic, R., & De Rosario, H. (2017). *pwr: Basic functions for power analysis.* [Computer software]. https://cran.r-project.org/web/packages/pwr/

Everitt, B. S. (1975). Multivariate Analysis: The Need for Data, and other Problems. *British Journal of Psychiatry*, *126*(3), 237–240. https://doi.org/10.1192/bjp.126.3.237

Krebs, G., Fernández de la Cruz, L., Monzani, B., Bowyer, L., Anson, M., Cadman, J., Heyman, I., Turner, C., Veale, D., & Mataix-Cols, D. (2017). Long-Term Outcomes of Cognitive-Behavioral Therapy for Adolescent Body Dysmorphic Disorder. *Behavior Therapy*, *48*(4), 462–473. https://doi.org/10.1016/j.beth.2017.01.001

MacCallum, R. C., Widaman, K. F., Zhang, S., & Hong, S. (1999). Sample Size in Factor Analysis. *Psychological Methods*, *4*(1), 84–99.

Mataix-Cols, D., Fernández de la Cruz, L., Isomura, K., Anson, M., Turner, C., Monzani, B., Cadman, J., Bowyer, L., Heyman, I., Veale, D., & Krebs, G. (2015). A Pilot Randomized Controlled Trial of Cognitive-Behavioral Therapy for Adolescents With Body Dysmorphic Disorder. *Journal of the American Academy of Child & Adolescent Psychiatry*, *54*(11), 895–904. https://doi.org/10.1016/j.jaac.2015.08.011

Rautio, D., Gumpert, M., Jassi, A., Krebs, G., Flygare, O., Andrén, P., Monzani, B., Peile, L., Jansson-Fröjmark, M., Lundgren, T., Hillborg, M., Silverberg-Mörse, M., Clark, B., Fernández de la Cruz, L., & Mataix-Cols, D. (2022). Effectiveness of Multimodal Treatment for Young People With Body Dysmorphic Disorder in Two Specialist Clinics. *Behavior Therapy*, *53*(5), 1037–1049. https://doi.org/10.1016/j.beth.2022.04.010

Schneider, S. C., Baillie, A. J., Mond, J., Turner, C. M., & Hudson, J. L. (2018). Measurement Invariance of a Body Dysmorphic Disorder Symptom Questionnaire Across Sex: The Body Image Questionnaire–Child and Adolescent Version. *Assessment*, *25*(8), 1026–1035. https://doi.org/10.1177/1073191116679504

Veale, D., Ellison, N., Werner, T. G., Dodhia, R., Serfaty, M. A., & Clarke, A. (2012). Development of a Cosmetic Procedure Screening Questionnaire (COPS) for Body Dysmorphic Disorder. *Journal of Plastic, Reconstructive & Aesthetic Surgery*, *65*(4), 530–532. https://doi.org/10.1016/j.bjps.2011.09.007
